# Isolated Sixth Cranial Nerve Palsy as Initial Presentation of Pituitary Apoplexy: A Case Report

**DOI:** 10.7759/cureus.41154

**Published:** 2023-06-29

**Authors:** Abdalla Fadul, Gokhan Demir, Mohammad Safieh, Ahamed Lebbe, Fatema Falamrz, Ahmed T Alhassan, ELMustafa Abdalla, Mohamed H Fadul, Mohamed Elawad, Raza A Akbar

**Affiliations:** 1 Internal Medicine, Hamad Medical Corporation, Doha, QAT; 2 Medicine, Hamad General Hospital, Doha, QAT; 3 Anaesthesia, Hamad Medical Corporation, Doha, QAT; 4 Faculty of Medicine, University of Khartoum, Khartoum, SDN; 5 Radiology, Hamad General Hospital, Doha, QAT; 6 Internal Medicine, Hamad General Hospital, Doha, QAT

**Keywords:** isolated cranial nerve palsy, diplopia, sixth nerve palsy, pituitary apoplexy, pituitary adenoma

## Abstract

Pituitary apoplexy is a serious condition, which if left untreated, might lead to irreversible life-long complications. Hence, pituitary apoplexy should always be included in the differential diagnoses of a patient with an isolated sixth cranial nerve (CN) palsy. This report highlights the case of a patient presenting with isolated CN palsy associated with pituitary apoplexy. Although pituitary adenomas are common, they seldom present with isolated abducent nerve palsy without any other CN involvement. The 47-year-old female patient presented with acute right eye pain, diplopia, and a squint. Examination revealed an isolated unilateral sixth CN palsy. Brain MRI showed a sellar and suprasellar mass suggestive of hemorrhagic pituitary apoplexy. The patient was transferred to neurosurgery and underwent transsphenoidal resection of a pituitary macroadenoma. Postoperative follow-up showed clinical improvement. It is, thus, imperative for physicians to have the knowledge to recognize an isolated sixth cranial nerve palsy and its associated causes.

## Introduction

Pituitary adenomas are common sellar masses with a prevalence of around 20% [1], especially after the third decade of life [2]. They usually present with different signs and symptoms. Pituitary apoplexy is an important complication of rapidly growing pituitary adenomas defined as acute hemorrhagic infarction of the pituitary adenomas, and usually results in sudden onset of headache, nausea/vomiting, and other different manifestations of increased intra-sellar pressure [3]. Among the common clinical manifestations are neuro-ophthalmic ones, including visual loss, visual field defects, ophthalmoplegia, and hypopituitarism [2,3]. These complications happen because of either chiasmal compression or lateral extension of the adenoma into the cavernous sinus wall (which mainly occurs with pituitary apoplexy) causing compression on the oculomotor nerve and, rarely, the abducent nerve [4-7].

We present a case of a young female who presented with an isolated right sixth cranial nerve (CN) palsy due to hemorrhagic pituitary apoplexy.

## Case presentation

A 47-year-old female patient presented to the emergency department (ED) complaining of acute onset of pain in her right eye for the preceding 24 hours. The following day, the eye pain resolved but she developed new onset double vision along with a squint in her right eye which she noticed while looking at the mirror. She denied any history of trauma. There was no history of any preceding or concurrent focal infective symptoms, vomiting, morning headaches, loss of consciousness or any jerky movements.

Her past medical history included end-stage renal disease (ESRD) for which she was on hemodialysis, hypertension, dyslipidemia, and parathyroid adenoma. She reported good compliance with her medications but did not keep a diary of her blood pressure readings.

On examination, she had normal vital signs. Her cardiovascular, respiratory and abdominal examinations were unremarkable. Her neurological examination revealed a normal power of 5/5 grade in all four limbs. Her sensory exam was normal. Her cranial nerve examination revealed a right-sided sixth CN palsy with conversion squint in her primary gaze. The other CN examination was unremarkable. Her clinical review by the ophthalmologist revealed painless, abduction deficit diplopia in the primary vision and right gaze. Her eye examination for optic discs and maculae was normal. She had no relative afferent pupillary defect (RAPD) and her colour vision was intact. The patient was admitted for workup for an isolated sixth CN palsy. The pituitary function was assessed, and laboratory results are illustrated in Table 1.

**Table 1:** Laboratory results of the patient. FSH: Follicle-stimulating hormone; LH: Luteinizing hormone; TSH: Thyroid stimulating hormone; FT4: Free thyroxine; IGF: Insulin-like growth factor; AM: Ante mridiem; ACTH: Adrenocorticotropic hormone.

Investigation	Patient value	Reference range
Prolactin	776	102-495 mIU/L
FSH	>200	26-135 IU/L
LH	98	8-59 IU/L
Estradiol	59.4	18-505 pmol/L
TSH	1.67	0.3-4.2 mIU/L
FT4	15.2	11-23.3 pmol/L
IGF	122	76-219 mcg/L
ACTH (AM)	58	7.2-63.3 pg/mL
Cortisol (AM)	416	138-689 nmol/L

**Figure 1:**
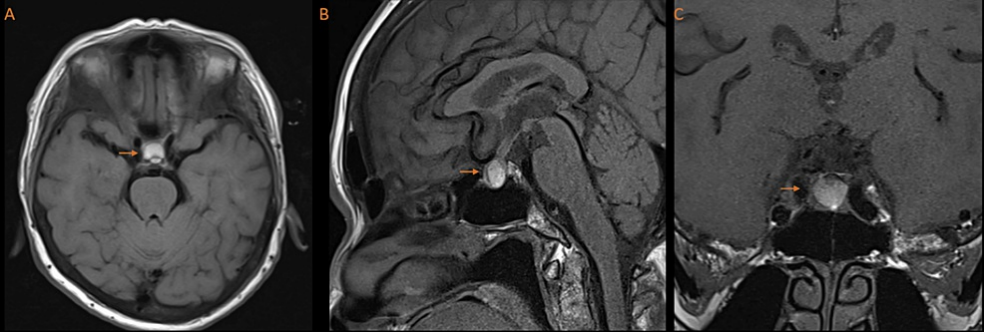
Axial (A), sagittal (B), and coronal (C) T1-weighted images of sellar/suprasellar oval-shaped homogenous hyperintense mass (arrows), effacing the suprasellar cistern, no cavernous sinus invasion, no definite optic chiasm compression.

She underwent a magnetic resonance imaging (MRI) scan of her brain and orbits (Figure 1), which showed chronic ischemia of the frontal white matter and old microbleeds in the left occipital lobe and right temporal lobe. There was a mass in the sellar and supra-sellar regions, which likely represented a hemorrhagic pituitary adenoma.

The patient was eventually transferred to the neurosurgical team, and she successfully underwent a transnasal, transsphenoidal resection of pituitary macroadenoma. The surgery was uneventful. Her diplopia and vision improved postoperatively. She was kept for observation for a few days and was then safely discharged with outpatient clinic follow-ups arranged.

## Discussion

We presented the case of a 47-year-old woman with isolated right 6th nerve palsy, found to have hemorrhagic pituitary adenoma based on brain MRI.

Isolated unilateral abducens nerve palsy (IUANP) is a rare condition that can be caused by various etiologies, including vascular, neoplastic, and inflammatory [8]. In some cases, IUANP can be a rare complication of pituitary apoplexy [9]. Pituitary apoplexy is a medical emergency that occurs when there is a haemorrhage or infarction of the pituitary gland, which can lead to compression of the adjacent structures, including the CNs. The presentation of pituitary apoplexy can be mistaken for subarachnoid haemorrhage, and MRI is the imaging modality of choice [9]. The abducens nerve runs medial to the third, fourth, and first two divisions of the fifth CN, all of which run in the lateral border of the sinus [10]. So, the abducens nerve is in a protected position in the sinus, which makes isolated abducens palsy rare.

There have been only a handful of cases of isolated sixth nerve palsy as a complication of pituitary apoplexy. One case reported in 2006 described a 68-year-old man with diabetes and hypertension who presented to the clinic with an isolated sixth nerve palsy [11]. However, the patient proceeded to develop a third nerve palsy two days later, before going into cardiac arrest, leading to his death. Another case reported was of a 44-year-old woman with a history of sarcoidosis who presented with an isolated sixth nerve palsy [5]. She had a rapidly expanding pituitary adenoma (without apoplexy) that was safely resected. Also, a case report described a 68-year-old male patient who presented with an isolated abducens palsy after mild head trauma and was found to have a pituitary adenoma. The bleeding tumour expanded rapidly to compress the abducens nerve [12]. 

The treatment of pituitary apoplexy includes high-dose corticosteroid administration and surgery [13]. Transsphenoidal surgery is indicated in patients with diminished levels of consciousness, hypopituitarism, or visual field defects [13]. In some cases, endoscopic transsphenoidal procedures can be used to resect the pituitary adenoma with hemorrhagic and necrotic changes, which can lead to prompt and complete improvement of the third nerve palsy [14].

IUANP can also be caused by neurovascular compression, which is a rare cause of the condition [15]. While most cases of IUANP experience spontaneous resolution, some require surgical treatment [15]. A close evaluation of the neurovascular relationship is necessary to determine the cause of IUANP [15].

## Conclusions

IUANP is a rare condition that can be caused by various etiologies, including pituitary apoplexy and neurovascular compression. The treatment of pituitary apoplexy includes high-dose corticosteroid administration and surgery, while IUANP caused by neurovascular compression may require surgical treatment. A close evaluation of the neurovascular relationship is necessary to determine the cause of IUANP. This case contributes to the current body of literature on reported cases with this unusual presentation.
